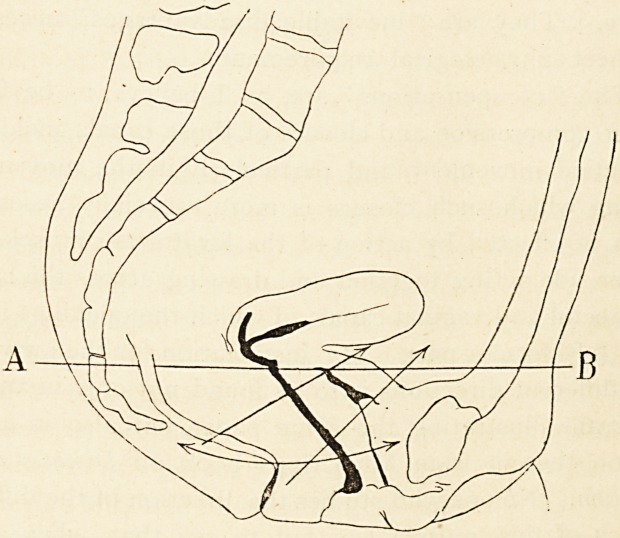# On Some Points in the Surgical Physiology of the Abdomen and Pelvis
**Resumé* of an unwritten address delivered to the Gloucestershire Branch of the British Medical Association.


**Published:** 1890-06

**Authors:** T. S. Ellis

**Affiliations:** Consulting Surgeon to the General Infirmary at Gloucester


### ON SOME POINTS IN THE SURGICAL PHYSIOLOGY OF THE ABDOMEN AND PELVIS.*

BY

T. S. ELLIS,

*Consulting Surgeon to the General Infirmary at Gloucester.*

---

PHYSIOLOGY, or—as it is still called in Scotland—the *Institutes of Medicine*, has been too much regarded as bearing only, or mainly, on medical practice. *Surgical Anatomy* is an expression very familiar; *Surgical Pathology* hardly less so; and even *Surgical Bacteriology* is the title of a book; but the words *Surgical Physiology* are now, so far as I am aware, for the first time linked together. And yet illustrations are abundant, literally from the head to the foot, of the close relation between the study of function and the practice of surgery. The surgery of the brain depends on accurate knowledge of the functions of that organ in its different parts. So, also, much of the wide domain of ophthalmic surgery is closely related to the functions of the eye. And, if I may be permitted to refer to work of my own, I have shown that deformities of the foot are due to failure of function, and that by renewal of it, in specially marked degree, restoration of form will result. It was my good fortune to receive my physiology at the hands of a surgeon: the teaching of Sir James Paget, in his lectures at St. Bartholomew's, has, I am sure, had a life-long influence on my habit of thought, more than I have at all times been conscious of.

* *Resumé* of an unwritten address delivered to the Gloucestershire Branch of the British Medical Association.

I think, too, that I might say this of a passage in the first book on medicine I ever read. Sir Thomas Watson, in his *Lectures on Physic*, puts among the objects of admiration to be found by contemplation of the human body, "its compensation for inevitable disadvantages." These "compensations" are, as I hold, much more often to be found in free exercise of functions than is generally recognised. It is manifest that, in order to put this free exercise of function into operation without risk of injury, full knowledge of the functions themselves is essential.

The "disadvantages," in respect of strength and stability, "inevitable" in a structure whose different parts move freely on each other, have, in the case of the human body, a "compensation" in that very mobility itself. The muscles which move, in moving sustain. By thus relieving the ligaments on which, in a static condition, the strain falls, an opportunity for recoil and recuperation in those structures is given. This principle, in my view, holds good in respect of the intestines. They are supported by ligament in the form of a mesentery: such support is liable to over-strain: where is the compensation? Mr. Lockwood, in his recent book on hernia, has forcibly directed attention to the "suspensory muscle" contained in the mesentery. Now, in hernia, as in my student-days Mr. Abernethy Kingdon used to insist, a relaxed state of the mesentery is a condition necessary for the protrusion. If, then, it be possible to stimulate the action of the suspensory muscle and so relieve the over-strained ligament, and, in doing so, to shorten it, we have an indication from a surgical point of view. For I take it that the law that muscles, strengthened by action, tend to remain firm and taut when not in use, and that ligament adapts itself to the length required, holds good in this as in any other structure. As the muscle is free from any direct control by the will, we can only look for some muscle or muscles, under control of the will, with which it is likely to be associated.

Before I discuss this question further, I would mention another form of prolapse, to which I might have hesitated to apply the term "hernia" if it had not already been so applied. Dr. Herman, in a very interesting paper,* has so designated prolapse of the uterus. It is, indeed, a hernia, a partial protrusion from the combined abdominal and pelvic cavity of that which should be completely within it; and, being a displacement, it is, essentially, a surgical condition. Dr. Herman replies to the important question—What supports the uterus?—"The pelvic floor supports it;" and adds, "This is mainly muscular, and the great muscle in it is the levator ani." Now, if we regard prolapse of the uterus as due to failure of its proper support, and if this support be the levator ani muscle, then we have in more vigorous action of that muscle an indication for treatment. We have little to hope for any action of it that could be directly induced by the will, and the question before us seems to me to be this:—Is there any movement under control of the will with which it is likely to be associated? There are parts, also liable to prolapse, on which the influence of this muscle is more apparent than it is as regards the uterus—the rectum and anus. Moreover, a persistently congested condition of these parts leads to thickening of the tissues and adventitious growth, to hæmorrhoidal tumours. Now, we know that muscular action is a powerful agent in promoting circulation and so relieving congestion. This suggests the question, whether more

* Hunterian Society Oration, *British Medical Journal*, June 1, 1889.

vigorous action of the levator ani might not have an influence on prolapse of these parts and on the painful congestions to which they are liable? Here, too, we have little to hope for any action under direct control of the will; and we must look for movements, under control of the will, with which such action is likely to be associated.

The importance of the levator ani muscle has, I am sure, been much underrated. In Quain's *Anatomy*, its action (with the coccygeus) is given as, to "elevate the lower part of the rectum and invert its anal border, after the protrusion and eversion which accompany defæcation." I would direct particular attention to it in relation to its line of action across the line of the rectal and vaginal canals. The diagram has been adapted from one in a very interesting paper "On the Rectum and Anus,"* by Mr. Symington. He forcibly points out the

* *Jour. Anat. and Phys.*, vol. XXIII.

importance of regarding the anal and vaginal canals as being closed, collapsed with the sides in contact, and not (as often shown in views of the pelvic organs) open tubes. This condition may, however, be true of the rectum, which, when not containing fæces, may be distended by air. I have drawn across the pelvis an imaginary line AB. The interruptions in that line indicating the rectum and vagina, each of them a distensible canal surrounded by yielding tissues, are, manifestly, elements of weakness in the structure regarded as a support for the parts above. They are "inevitable disadvantages" necessary to meet physiological requirements.

The "compensations" are, as I believe, to be found in the compression and closure of those canals incidental to active movement, and particularly in the movements during which such closure is more especially necessary. This is effected by action of the levator ani muscles, on either side acting together and drawing across the line of the rectal and vaginal canals of which the openings in the line AB form a part. My justification for the arrows in the different directions is to be found not only in another diagram illustrating the same paper, but also in a quotation therein from Mr. Cripps' book on *Diseases of the Rectum*. No one who studies the direction of the different fibres of this muscle can fail to see that, when acting simultaneously on both sides, they "will act powerfully as compressors of the rectum." But, in doing this, they must also compress the vagina. The need of this compression is obvious; for, if it remained, during active motion, in the same relaxed condition as found in the recumbent, flexed position, no one could believe that the ligamentous attachments of the uterus would be at all adequate to prevent it from slipping down the distensible vagina. I do not, myself, believe that ligamentous tissue alone is ever sufficient for the support of any part or organ. Relief, at least occasional, by muscular action, is everywhere necessary. In the case before us it is afforded by the levator ani.

The position of rest, which I discussed before this Branch twelve years ago, is, speaking generally, one of flexion. I then defined it as that of least tension and of least pressure, where the position of the limbs is a mean between the extremes of motion. The flexed or squatting position is that in which the rectum best allows the contents of the intestine above to pass downwards and itself to be evacuated. This flexion may, for this purpose, be extreme, but extension is incompatible with it. The flexed position is that in which the vagina is most relaxed, as shown in the parturient woman and in examinations of the uterus. If, therefore, it be true that the function of the levator ani muscles, acting with the sphincters, is to lift upwards and to support the pelvic viscera, and to close the pelvic outlets, it is not likely that such action would be associated with the act of assuming the flexed or squatting position. It is more likely to be associated with the act of vigorously springing to extreme extension. To do this fully and repeatedly it is necessary that flexion should alternate with extension, complete relaxation with extreme tension. This is necessary in order that movement to the extended position shall take place in the most vigorous manner. Such movement is always synchronous with the respirations; the inspiration always going with the upward, the expiration with the downward movement.

Anyone who has watched a woodman felling a tree will confirm this. In such work the tool is thrown upwards, dragging on the arms as in the Sylvester method of artificial respiration, which thus aids the inspiration. The lungs are fully inflated, inviting, so to speak, the blood from the right side of the heart. The diaphragm, in descending, relieves from strain the ligamentous attachments of the liver, at the same time promoting, by pressure, the onward flow of blood from that organ, which, in turn, is re-supplied by blood from the other abdominal and pelvic viscera. At the same time onward circulation from the lower limbs is promoted by the vigorous muscular action of raising the body and sustaining it for the downward blow. We have here a pretty good illustration of a point always strongly enforced, in his lectures, by Sir James Paget—that we must, in the living body, look for more than one result from any exercise of function. My suggestion is, that with every one of these upward movements, the intestines, the uterus (in the case of a woman), the rectum and anus are, all of them, braced up and tendency to prolapse prevented, by action of the levator ani and sphincter muscles; and that action of the suspensory muscle bracing up the intestinal support is also attendant on each inspiration. The exercise has seemed to me to involve the greatest amount of beneficial influence, mechanically, on the supports of the abdominal and pelvic organs, and, physiologically, on the circulation in them and throughout the body as a whole, as well as promoting the fullest, because the deepest, respiration. Seeing that all this is done without any undue strain on the heart—for the blood is in every way helped onwards—and that it involves no excessive muscular exertion, we need not be surprised that Mr. Gladstone feels himself the better, even at his age, for engaging in such exercise. It involves a vigorous "reaching upwards," which, as I

have said elsewhere,* is "a good thing to do, not only in a metaphorical sense."

Why, in the exercise described, does the inspiration go with the upward movement? and why should the contraction of the levator ani and of the suspensory muscle of the mesentery be supposed to be also attendant on the inspiration? The greatest downward pressure on the abdominal and pelvic organs occurs as the diaphragm descends with the deepest inspirations: then the greatest need exists for closing the pelvic outlets, and so preventing prolapse. With the expiration the abdominal organs are, so to speak, invited upwards to fill the hollow caused by the increased arch of the ascending diaphragm: then there is lessened downward pressure, and consequently less risk of prolapse, less need for closing the pelvic outlets. Contraction of the abdominal muscles is not needed for the upward spring: it is essential to the downward blow. But if the descent of the diaphragm and the contraction of the abdominal muscles were simultaneous, the double pressure on the abdominal and on the pelvic organs, and, through them, on the several outlets, would be excessive. The tendency to prolapse would be much greater, because there would be a persistence of the conditions which exist during efforts of defæcation—conditions which are well known to involve a tendency to hernia or prolapse.

Various considerations point to the probability that the suspensory muscle of the intestine acts with the inspiratory rather than with the expiratory movement, and only slightly, if at all, in any but deep inspiration. Under ordinary conditions there is little downward pressure by the diaphragm on the contents of the abdomen, and these

* *The Human Foot*, page 83.

(the abdominal muscles being relaxed) are free to bulge forwards. In very deep inspirations there is great downward pressure, and the abdominal muscles, though not in action, are drawn upwards by the uplifted ribs and are so rendered tense, thus preventing the abdominal organs from bulging forwards. This is especially the case if there be attendant springing upwards to extreme height, and the more if the arms be also thrown upwards. All this takes place in the exercise under consideration. In full expiration, even if sudden as in coughing, there cannot be any great strain on the supports of the abdominal contents; the abdominal muscles follow on and sustain them as they move upwards with the diaphragm. Not so in deep and, especially, in sudden inspiration. It is true that the liver, lying closely beneath the diaphragm and attached to it in front and behind, moves downwards with it; there can be no strain on the ligamentous support of that organ. With the intestines the case is, however, different; they are attached to the pillars of the diaphragm which do not move downwards, while they are acted on by the downward pressure of the expanded portion which does so move. Thus, while an ordinary inspiration may not involve sufficient movement to cause any strain, a sudden and deep inspiration might cause undue stretching of the mesentery if no provision against it existed. The "compensation" for this apparently "inevitable disadvantage" is, I believe, to be found in the suspensory muscle, which I regard as the provision for relieving the suspensory ligament or mesentery from strain.

This view seems to me to accord with facts coming within our knowledge. It is with the expiratory rather than with the inspiratory effort of coughing that we feel the protrusion into the hernial opening of an incipient hernia. Unless there be some counteracting influence, why is it that the protrusion is not most felt when there is greatest downward pressure? This must be in the deep inspiration which precedes the cough. It has, moreover, seemed to me remarkable that excessive coughing does not tend to the production nor even to the aggravation of existing hernia nearly so much as might have been expected. Lifting heavy weights and straining efforts at defæcation are much more frequent causes. A case is reported in the *Lancet* of October 5th, 1889, where a hernia long strangulated which had resisted efforts at reduction was, at length, reduced during a cough, and, apparently, by means of it. I could not accept the explanation given: to me it seemed more likely to be due to a contraction of the suspensory muscle. I have noticed in examining boys for hernia that the cremasters act, on coughing, with the inspiration and not with the expiration.

All the facts and considerations arising therefrom, as they present themselves to me, seem to justify the induction of some such laws as these:

(1) Exercises which involve alternate springing upwards to extreme extension, and sinking downwards towards the squatting position (such double movement being associated with full inspiration in springing upwards and full expiration in sinking downwards), tend to cause the most vigorous action of the muscular agencies which support the abdominal and pelvic organs, promoting free circulation in them, and to close the pelvic outlets.

(2) Exercises which involve effort during the maintenance of the flexed or squatting position, or which interfere with free respiration, tend to increase the strain on the supports of the abdominal and pelvic organs and to prevent free circulation in them.

In this I find indications not only for the prevention but for the treatment, with a view to cure, of all of the prolapsed and congested conditions which constitute the several surgical affections mentioned. If it be true that muscles strengthened by use tend to remain firm and taut when not in use; if it be true that the levator ani in action does lift up and compress the rectum and vagina, promoting circulation in, around those parts, and that in sudden and deep inspirations the suspensory muscle of the mesentery does brace up and shorten the ligamentous attachment of the intestine, then, such conditions being fulfilled in the exercise described, the probability is, I think, fairly deducible that such exercise would from the surgical point of view be beneficial.

But, it may be fairly asked, do results justify the deductions made? So far as my experience goes, they do. I have certainly seen cases where hæmorrhoids and the various forms of prolapse mentioned have got well under treatment by special exercise alone. When one induces a law, and deduces a probability from it which, when tested, is followed by the expected result, one naturally regards it as cause and effect. But if I could bring any such number of cases as could in the experience of one man be collected, and if my assurance that no other cause had operated in producing a cure were ever so fully accepted, that alone would not prove my case. We know that these morbid or defective conditions do disappear, and we do not always know why. The recovery is put down to improved health or some other indefinite cause. The question, what forms of exercise will be of benefit and what forms will do harm in a case, say of hæmorrhoids, is often of urgent importance. I know of no recognised principle by which it could be decided. Nor do I see how any collection of facts, at hazard collected, is likely to decide it. By the "imagination of possible truth," and by testing whether it were reliable, much real and valuable truth has been reached. I think that it is the author of *Rab and his Friends* who has somewhere said that "the human intellect, with a dog-like instinct, hunts best when it has scent." It is for you to say whether my utterances involve a "possible truth" worthy to be tested; whether it be worth while to follow up the "scent" I have endeavoured to show. Permit me to recall the time, fifteen years ago, when I told, at a meeting of this Branch, the story of my own flattened foot, the result of an accident six years before; how it had been cured by exercise, on principles which I endeavoured to indicate. I was, by authority, then told that it could not be, that I must have got well from some other cause. I have lived to see the principle accepted and applied as a standard method of treatment in almost every part of the world. It may seem to be a far cry from flat-foot to hæmorrhoids, or prolapse of intestine or of womb—Dr. Herman has actually compared this last to flat-foot. And, indeed, are they not all instances of relaxation become permanent as a consequence of defective "compensation"? Not without deliberation, but withal in full confidence, I make the prediction now that the time will come when all these conditions will be treated, not by rest and "recumbency," except so far as it is an aid to the more potent agency of well-directed vigorous exercise; when we shall no longer see "recumbency" put first in a list of "remedies which diminish congestion of the pelvic organs," followed by others, none of them having any reference to the physiological agencies which promote free circulation—I quote the words of one from whom I had been led to expect better things. Two facts only I will state now. The worst and most persistent case of piles I ever knew was in a blacksmith. Here was a case of a healthy man, accustomed to regular, hard, muscular work; yet, although he was of strictly temperate habits, and lived, otherwise healthy, to very old age, he had a malady which is usually associated with sedentary habits. Now his work involved the maintenance of a stooping, semi-squatting position all day. If it could be shown that strikers, who wield sledge-hammers, were specially liable to piles, it would be fatal to my theory. Dr. Soutar, of Barnwood House, has told me of a fellow-student at Edinburgh who, having piles badly, took to throwing the hammer as an exercise, and they speedily disappeared. This is as, theoretically, it should be.

If it be asked whether the subject before us is not rather hygienic than strictly surgical, my answer is, that the scientific surgeon ought to be something more than a mere hand-worker (chirurgeon); he should rather be regarded as one existing for the cure of surgical ailments. John Hunter, whose authority will be recognised, said, in speaking of cancer: "For what I call a cure is an alteration of the disposition, and of the effects of that disposition, and not the destruction of the cancerous parts."* Now we pad, by means of trusses, hernial openings; and prop up, by means of pessaries, prolapsed uteri: but we cannot call such proceedings cures. Nor, as it seems to me, do the so-called radical cures go to the root of the matter. If, too, by constriction or by cutting operation, we bring about a complete "destruction" of a hæmorrhoidal mass or prolapsed rectum; however satisfactory

* Quoted by Sir Spencer Wells in the Morton Lecture.

the result appear, we have no right, on the Hunterian definition, to speak of it as a cure at all. But if the "disposition" be due to defective function, and if by restoring function we remove it, with "the effects of that disposition"; then, as I claim, the definition of a cure is fulfilled.

To the objection that a speedy result is by patients demanded, and that treatment by physiological means would not be accepted because too slow, the answer is, that the necessary seclusion which an operation requires, added to that required for recovery from it, is often a great loss of time. Add to this the interference with health, and the not altogether imaginary risk, and strong inducements still exist for the adoption of treatment other than operative. Moreover, no one suggests that operations can be superseded altogether. Inasmuch, however, or, at least, in so far as we do nothing to "alter the disposition," but only remove the "effects," they do not really result in cures.

The cure of hernia by exercise, although ignored by writers in this country, is by writers on the Swedish movement cure alleged to result from it, but the explanation differs from that which I have given. In *Home Gymnastics*, by Professor Hartelius (translated by Miss Löfving), he states that the "scientific explanation" is "that, by certain movements, the muscles which surround the rupture are strengthened and increased in bulk so as to contract the passage." I cannot accept this as sufficient, although, undoubtedly, the enlarged inguinal canal is diminished by action of the abdominal muscles. It does not, however, remain closed, or even nearly so, during rest, even where the action is habitually vigorous and the development well marked. I am, as already indicated, much more disposed to believe in the association of these voluntary muscles and of those concerned in respiration with the involuntary suspensory muscle of the mesentery. Both of these have a close association with the peristaltic action of the intestines.

How far a want of "accurate and consentaneous physiological harmony in these co-operating structures" —I quote these words from *Hilton on Rest*—may account for some of the "entanglements" which have, in some cases, been discovered to be the only explanation of peritonitis and intestinal obstruction, is a question of much interest. Is it not possible that a restoration of that harmony may account for the sudden yielding of symptoms otherwise unaccounted for—that the displaced intestines may be restored to proper position by action of the muscular element which, as I contend, is necessary to maintain that position? If this be conceded as possible, then all that concerns this "physiological harmony" comes within the province of the surgeon, and the question, What agency will best promote it? is of great practical importance. The proper place, in treatment, of such agencies as warmth, moisture, dry friction, muscular action, massage, and galvanism—faradic and continuous—calls for study more careful than, so far as books reveal it, has yet been given. *Abdominal Surgery* has come too much to signify *the operative surgery of the abdomen.* It will not be to the credit of our art if the brilliant results in that department attained should divert attention from the study of physiological laws and processes, a better knowledge of which may more often result in the success of other treatment, and render operative interference unnecessary.

## Figures and Tables

**Figure f1:**